# Color Compounds Removal from Tequila Vinasses Using Silica Gel Adsorbents Functionalized with Thiol Moieties: Equilibrium and Kinetics Studies

**DOI:** 10.3390/molecules29245910

**Published:** 2024-12-15

**Authors:** Carlos Gonzalez-Valerio, Alejandro A. Peregrina-Lucano, Ricardo Manríquez-González, Aida A. Pérez-Fonseca, Jorge R. Robledo-Ortíz, Ilya G. Shenderovich, Sergio Gómez-Salazar

**Affiliations:** 1 Chemical Engineering Department, University of Guadalajara—CUCEI, Blvd. Marcelino García Barragán #1421, esq. Calzada Olímpica, Guadalajara 44430, Jalisco, Mexico; carlos.gonzalez0543@alumnos.udg.mx (C.G.-V.); aida.perez@academicos.udg.mx (A.A.P.-F.); 2 Pharmacobiology Department, University of Guadalajara—CUCEI, Blvd. Marcelino García Barragán #1421, esq. Calzada Olímpica, Guadalajara 44430, Jalisco, Mexico; aapl69@hotmail.com; 3 Department of Wood, Cellulose, and Paper, University of Guadalajara—CUCEI, Zapopan 45150, Jalisco, Mexico; ricardo.manriquez@academicos.udg.mx (R.M.-G.); jorge.robledo@academicos.udg.mx (J.R.R.-O.); 4 Faculty of Chemistry and Pharmacy, University of Regensburg, Universitaetstrasse 31, 93053 Regensburg, Germany

**Keywords:** tequila vinasses, sol–gel, silica gel, adsorbent, adsorption

## Abstract

Tequila vinasses are organic wastes generated during ethanol fermentation at elevated temperatures (≥90 °C) and pH ≤ 4.0, making them hazardous to the environment. This paper describes a new, simplified UV–vis spectroscopy-based procedure for monitoring the adsorption of color compounds in tequila vinasses onto silica-based adsorbents, along with an optimized synthesis method to produce the most efficient sol–gel synthesized thiol-functionalized adsorbent. Under optimized conditions, the uptake capacity of this adsorbent reaches 0.8 g g^−1^ in 90 min. Experimental results demonstrate that the adsorbent has a specific affinity for melanoidin-type molecules. The adsorbent demonstrates excellent thermal stability (~316 °C). The results of this work indicate that the adsorbent possesses potential in the treatment of tequila vinasses from wastewater discharges.

## 1. Introduction

Tequila is the most famous Mexican spirituous drink worldwide, produced from the brewed must of an assortment of *Agave tequilana var. Azul*. The production of tequila generates wastes called vinasses. These wastes are generated by the tequila industries from the residual solution of fermented agave sugars after alcohol distillation. It has been reported that about 10–12 L of vinasses are generated by each liter of tequila produced [1]. Tequila vinasses (ca. 80%) are mostly discharged to water bodies and agricultural lands with almost null treatment representing a serious environmental risk [2]. Specifically, tequila vinasses constitute a sort of wastewater possessing high degradation strength and, consequently, a high degree of pollution since they are discharged at elevated temperatures (≥90 °C), pH ≤ 4.0, and very high chemical oxygen demand (COD, 5–150 g L^−1^) [1,2,3].

The treatment of tequila vinasses wastes becomes complex due to the contained compound composition and the large amounts generated. The compounds that grant the dark brown color to tequila vinasses are called melanoidins. It is well known that melanoidins are color compounds made up of aromatic heterocyclic structures with ionic moieties that are difficult to degrade by microorganisms; hence, biological treatments, in general, present bad yields, and combined waste treatments must be implemented to improve degradation efficiencies [4]. Amongst the processes used to treat these wastes are physicochemical, coagulation, sand filtration, Fenton oxidation, and membrane filtration [5]. These processes have proved to have high efficiencies for turbidity, and a chemical oxygen demand (COD) removal of up to 70% and 80%. However, they present high operational costs and high demand for reagents, making them less appealing. In contrast, the easy use of functionalized adsorbents makes this process a suitable technology to solve the problem presented by the tequila vinasses discharged into the environment [6]. For instance, there are some reports which indicate that waste vinasses have been used as raw material to obtain activated carbons and applied in dye removal and other components [7,8]; this is attributed to the phenolic and ionic nature present in the carbons. Another example of this methodology was presented by Caqueret et al. [9], who utilized activated carbon to adsorb compounds present in beet vinasses.

Tequila vinasses constitute a complex mixture of water, fermentable sugars, organic acids, esters, alcohols, furans, and phenolic compounds [10]. A current and reliable method of analysis and a quantification of all the components of tequila vinasses is not available yet. As a result, in most tequila vinasses, the unambiguous identification of all the compounds present is far from being a reality. This situation makes it difficult to identify the compounds of tequila vinasses in real samples. The traditional methods of analyzing vinasses use gas chromatography–mass spectrometry (GC-MS) or liquid chromatography (LC) techniques. For example, Lorenzo-Santiago et al. [10] drew upon a set of analytical methods which included ultra-performance liquid chromatography (UPLC) and ultra-performance liquid chromatography mass spectrometry (UPLC-MS) techniques to analyze some compounds (furans and phenolic) and some sugars, respectively, of tequila vinasses from Amatitan, Jalisco, Mexico. However, many tequila vinasses compounds, such as a significant amount of melanoidins, were not analyzed. In another study, Govea-Paz et al. [11] evaluated total sugars, total reducing sugars, and total phenols following the phenol–sulfuric, Miller, and 4-aminoantipyrine standard methods [12,13] when they studied the generation of biofuels using tequila vinasses in a continuous stirred-tank reactor (CSTR) with biomass carriers and an upflow anaerobic sludge blanket reactor (UASB) connected in series. Their results indicated that feeding more concentrated vinasses produced a decrease in hydrogen and methane production, coinciding with a 5 g/L-peak in reducing sugars. These studies indicate the complexity of analytical techniques that must be used to obtain satisfactory results about the tequila vinasses analysis. Based upon these results, in this work, a novel and relatively simple procedure of tequila vinasses analysis is proposed. The procedure involves a full UV-vis scan of tequila vinasses samples whose signals are further related to the drained masses of vinasses to construct the calibration curve of the weight of solids/mL vs. total absorbance. Then, to obtain the corresponding absorbances of all compounds present in the tequila vinasses for each point of the curve, a full UV-vis scan was performed in the range of 350–900 nm, followed by the numerical integration of the whole spectrum using Simpson’s 1/3 rule.

To the best of our knowledge, the use of functionalized silica gel adsorbents in treating tequila vinasses wastes has not yet been reported. The novelty of this work includes reporting on the synthesis and application of our newly prepared thiol-functionalized adsorbent in the treatment of tequila vinasses for the first time. Also, the novelty includes the proposal of a new analytical method to assess the tequila vinasses concentrations that is simpler and more robust compared to the conventional ones such as UV-vis or HPLC techniques. So far, no adsorbents of these types have been applied in the treatment of tequila vinasses, nor in the newly proposed analytical method. Using these adsorbents, it is expected to obtain improved removal efficiencies, fast removal kinetics, and substantial improvement in color compounds removal. This work aims to report on the use of a thiol-functionalized silica gel adsorbent to remove tequila vinasses color compounds. A simple analysis procedure of tequila vinasses was proposed, and the adsorption features of real tequila vinasses samples were studied through adsorption equilibrium and kinetic measurements. Various mathematical models were tested with experimental data to elucidate the adsorption mechanism of tequila vinasses on the adsorbent, and the rate-controlling mechanism in the kinetic measurements. Finally, the solid adsorbent was characterized by mass spectrometry, N_2_ adsorption, Fourier transform infra-red (FTIR), thermogravimetric analysis (TGA), and solid-state NMR techniques to gain insights concerning the obtained solid features.

## 2. Results and Discussion

### 2.1. Synthesis of the MaMPTXX Adsorbents

Several trials were performed to obtain an optimum adsorbent with enhanced characteristics such as minimal water solubility, maximized removal capacity of tequila vinasses compounds, and improved ligand densities. The tests consisted of varying the synthesis parameters, including the molar ratios of the functional precursor (FP) to the cross-linking agent (CLA) and the amount of stabilizing agent (NaCl), as shown in Table 1.

### 2.2. Adsorption Kinetic Results

**Figure 1:**
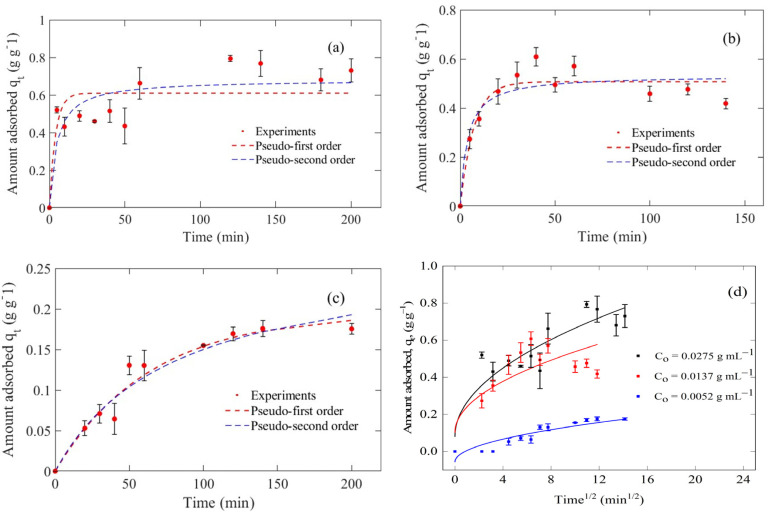
Results of adsorption kinetics of tequila vinasses compounds on the MaMPT002 adsorbent at three different initial conditions and fitting to three kinetic models. (**a**) C_o_ = 0.0275 g mL^−1^, (**b**) C_o_ = 0.0137 g mL^−1^, (**c**) C_o_ = 0.0052 g mL^−1^, (**d**) IPD model; symbols are experimental data, continuous lines are IPD model predictions.

The amount of tequila vinasses adsorbed on the MaMPT002 adsorbent as a function of time is shown in Figure 1 for the three initial tequila vinasses concentrations studied of 0.0052, 0.0137, and 0.0275 g mL^−1^. These concentrations were chosen to use an excess of thiol functional groups on MaMPT002 adsorbent and, simultaneously, to ensure adsorbent saturation. The experimental results suggest that it is the initial concentration that defines the adsorption rate, as higher concentrations lead to faster adsorption. From this perspective, the adsorption interaction between tequila vinasses color compounds and the MaMPT002 adsorbent is driven by the propensity of thiol groups to engage in interactions with compounds in the tequila vinasses. In the cases of the two highest concentrations, the equilibrium is attained after ~60 min, whereas in the case of the lowest concentration, the equilibrium is reached after ~140 min. These results reveal that the MaMPT002 adsorbent possesses improved kinetics and makes it appealing for tequila vinasses wastewater treatment applications.

Knowing the adsorption rates of the solid pellets is of paramount importance and integral to the adsorption of tequila vinasses color compounds. Several steps are generally recognized to be involved in the adsorption rate, from the fluid phase to the specific adsorption sites located at the interior of the pellets, and these include the following: (i) interplay with adsorption sites and (ii) diffusion to these sites. Three popular models were tested to ascertain the rate-controlling mechanism of tequila vinasses compounds on the MaMPT002 adsorbent, namely the following: (a) pseudo-first order (PFO), (b) pseudo-second order (PSO), and (c) intraparticle diffusion (IPD) (see Supplementary Materials for model descriptions). The experimental data were tested against each model to extract the corresponding parameters, and the results were used to find the best-suited model to the data. The results of fitting the kinetic models are shown in Figure 1a–d, and the values of the regressed parameters are in Table 2. These parameters demonstrate that the PSO model inadequately simulates the data, as the values of determination coefficients R^2^ for the three concentrations are significantly lower than unity, and the maximum adsorption capacities, *q_max_*, are not close to the experimental values. The PFO model is observed to match the experimental data better since the values of R^2^ are closer to unity and the *q_max_* values match the experimental values better. This model indicates that a reversible reaction occurs between tequila vinasses compounds and the thiol surface groups of the adsorbent, and a dynamic equilibrium linking the two phases is set in the adsorption operation. The values of the kinetic parameter of this model (*k*_1_) increase as the tequila vinasses concentration increases due to increased molecular collisions. On the other hand, the PFO and PSO models are not able to predict the mass transfer mechanism caused by diffusion. Because of this, the intraparticle diffusion model (IPD) was tested with kinetic data. A poor fitting between the model and the data can be seen in Figure 1d, which demonstrates that the adsorption of tequila vinasses compounds does not conform to the intraparticle diffusion mechanism. These results demonstrate that the adsorption kinetic data of tequila vinasses more closely follow a PFO model and indicate that the chemisorption is the limiting step of the adsorption rate of tequila vinasses compounds onto the MaMPT002 adsorbent.

### 2.3. Adsorption Equilibrium Results

#### 2.3.1. Effect of Tequila Vinasses Solution pH on Adsorption Extent

**Figure 2:**
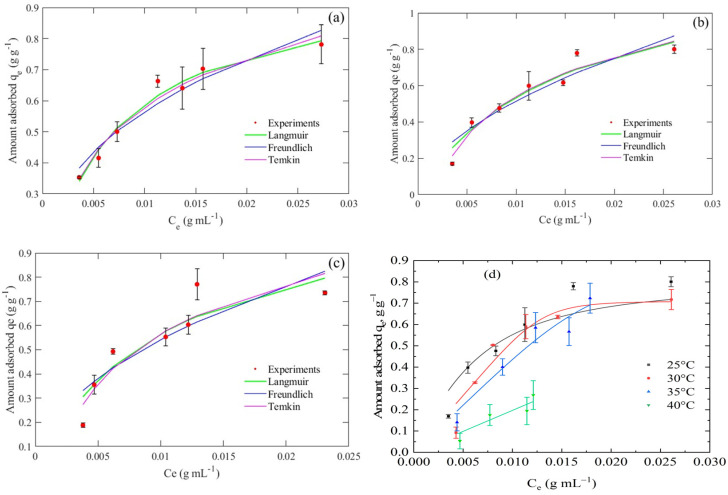
Experimental adsorption isotherms of tequila vinasses on the MaMPT002 adsorbent and simulations using three common isotherm models at three different pH values. (**a**) pH = 3.0, (**b**) pH 03.55, (**c**) pH = 4.0. (**d**) Effect of temperature on tequila vinasses uptake on the MaMPT002 adsorbent at three different temperatures.

The capacity of the MaMPT002 adsorbent on the tequila vinasses compounds removal was appraised at three different pH values (3.0, 3.55, and 4.0), distinctive of tequila industry wastewater discharges [14]. Figure 2 shows the tequila vinasses adsorption isotherms on MaMPT002 at 298 K. It is evident from Figure 2a–c that the pH has a slight effect on the tequila vinasses removal capacities as the maximum amounts at all pH levels change only slightly. At pH 3.55, the effective adsorption q_e_ increases from 0.16 g g^−1^ to 0.801 g g^−1^ as the equilibrium solution concentration C_e_ changes from 0.0035 g mL^−1^ to 0.026 g mL^−1^.

An overall picture of the tequila vinasses adsorption mechanism on MaMPT002 has been acquired by the direct application of several adsorption models (see Supplementary Materials). Figure 2a–c show the fitting results of experimental isotherms, and Table 3 depicts the values of the regressed parameters of these models. In all models, the determination coefficient R^2^ was used as a criterion to assess the goodness of fit. The Freundlich model is unacceptable because the R^2^ values are far from unity and it predicts adsorption capacity values, (q_e_), that are not close to the experimental data, especially at the lowest pH value (Figure 2a). In the case of the Langmuir model, despite the highest values of R^2^, this model cannot predict satisfactorily the values of q_max_, also at the lowest pH value (Figure 2a) and is unsatisfactory. In contrast, the Temkin model provides a quite accurate simulation of q_e_ at all three pH values, with R^2^ values close to unity.

**Scheme 1:**
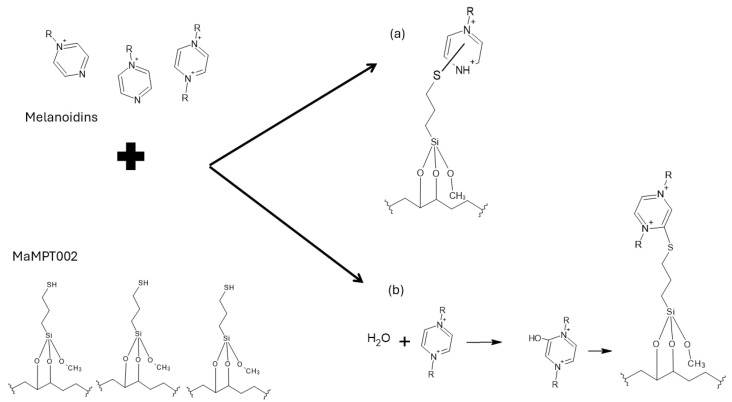
Proposed adsorption mechanism scenarios of color compounds (e.g., melanoidins) from tequila vinasses on the MaMPT002 adsorbent: (a) undefined thioether bond position in pirazinium, and (b) by the thioether bond in 2 ring position [15].

The results of these experiments allowed us to study the interactions between the predominant color compounds present in the tequila vinasses of the melanoidin-type (see Section 2.4), and the thiol functional groups of the MaMPT002 adsorbent. In this regard, it has been reported that the thiol groups can react with melanoidins through a covalent bond via a heterocycle that involves redox reactions through Maillard-derived pyrazinium compounds formed as byproducts of the oxidation of the pyrazinium radical cations [15]. A possible tequila vinasses uptake mechanism by the MaMPT002 adsorbent is shown in Scheme 1. In this mechanism two paths are proposed to occur, (a) in the presence of the nucleophilic thiols of the MaMPT002, melanoidins react to form the melanoidin-thioether bond (undefined position), (b) in a previous step, melanoidins react with water to form pyrazinium compound (such as the 2-hydroxy1,4-dihydropyrazine) which then react with the thiol groups of MaMPT002 to form the corresponding melanoidin-thioether bond via pirazinum ring.

#### 2.3.2. Comparison of Different Effluent Adsorption Performance of the MaMPT002 Adsorbent with Different Adsorbents

For comparison, Table 4 shows the adsorption capacities reported in the literature of the selected adsorbents applied in removing several types of vinasses based on the maximum amount of vinasse removed for each adsorbent (q_max_) and their specific surface areas. It is noticed that the two activated carbons (Picachem 150 and Picachem 120 PN) performed better than the bagasse fly ash but worse than both the peanut shell and the MaMPT002 adsorbent. Despite their higher specific surface areas, low vinasse compounds removal performance is also notorious in these carbons. These values suggest that the surface area is not a determining factor in the quantity of vinasse removed and does not confer high uptake efficiency.

Alternatively, the MaMPT002 adsorbent possesses a high specific surface area (432.68 m^2^ g^−1^), characteristic of mesoporous silica gel adsorbents synthesized by the sol–gel method. Moreover, the MaMPT002 adsorbent shows a similar vinasse removal capacity but with a simpler synthetic procedure than a peanut shell adsorbent. Finally, the thiol moiety in the MaMPT002 adsorbent confers a versatile alternative in the removal of other complex systems, including several heavy metal ions such as mercury [16,17], and lead [18]. The use of the sol–gel method for the synthesis of silica-based adsorbents allows obtaining materials with a predetermined large pore diameter and a significant internal surface area. Even pure silicon materials of this type significantly affect the mobility of adsorbed molecules due to various non-covalent interactions and hydrogen bonding [19]. Although the energy of such non-covalent interactions is low and they are unstable under ambient conditions, they still determine the average conformation of the partners [20]. Moreover, the adsorption capacity of such materials can be selectively and adaptively significantly increased by the chemical functionalization of their internal surfaces [21,22,23].

**Table 4:** Comparison of vinasse uptake using adsorbent MaMPT002 with different materials.

Adsorbent	Effluent Removed	S_BET_ (m^2^ g^−1^)	q_max_ (g g^−1^)	Reference
Picachem 150	Sugar beet vinasse	671.0	0.041	[9]
Picachem 120 PN	Sugar beet vinasse	634	0.1	[9]
Peanut shell	Sugarcane vinasse	40.71	0.562, 0.796	[24]
Bagasse fly ash	Sugarcane vinasse	−	0.017	[25]
MaMPT002	Tequila vinasse	432.68	0.801	This work

#### 2.3.3. Effect of Tequila Vinasses Temperature on Adsorption Extent

The effect of tequila vinasses temperature on the adsorption extent of the MaMPT002 adsorbent was investigated at three temperatures (25, 30, and 40 °C) and a pH of 3.55 using optimal conditions. It is apparent from Figure 2d that the removal capacity at 25–35 °C undergoes minor changes; however, it decreased as the temperature increased to 40 °C. The decrease in removal capacity demonstrates that the nature of the adsorption process was exothermic and chemical [26].

The thermodynamic variables standard free energy change ($∆G^{o}$), standard enthalpy change ($∆H^{0})$, and standard entropy change $\left(∆S^{o}\right)$ for the adsorption of tequila vinasses compounds on the MaMPT002 adsorbent, were determined by using the Van’t Hoff equation in the non-linear form as follows:

(1) $$K_{c}=\;e^{\left[\frac{{∆S}^{o}}{R}-\left(\frac{{∆H}^{o}}{R}\right)\frac{1}{T}\right]}$$

By the third principle of thermodynamics, $∆G^{o}$ can be calculated by the following:

(2) $$∆G^{o}=\;∆H^{o}-T\;∆S^{o}$$

where R is the universal gas constant (8.314 J K^−1^ mol^−1^), T is the absolute temperature, and K_c_ is the dimensionless thermodynamic equilibrium constant. The dimensionless form of K_c_ was obtained from the following definition [27]:

(3) $$K_{c}=\;\frac{1000\times K_{t}\times molecular\;weight\;of\;tequila\;vinasses\;\times tequila\;vinasses\;concentration}{\gamma }$$

where γ = tequila vinasses activity coefficient = 1 for a diluted solution; K_t_ represents the best isotherm model fitted constant (e.g., K_t_, the Toth equilibrium constant), determined for each dataset corresponding to each temperature. Equation (1) was used to obtain the thermodynamic parameters $∆H^{o}$ and $∆S^{o}$ by non-linear regression of the data using the Levenberg–Marquardt algorithm. All calculations were conducted using the Origin V9.8 2021^®^ software.

**Figure 3:**
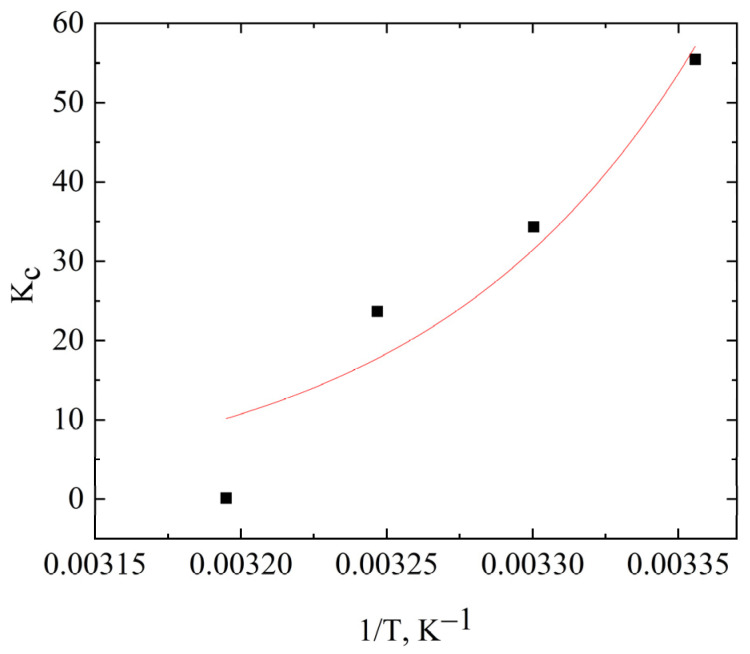
K_c_ as a function of the inverse of temperature and the non-linear fitting to Equation (4).

Figure 3 shows the results of non-linear fitting where the values of the Toth model constants, K_t_, were used for the calculations since this model was the one that best predicted the data for all temperatures. Table 5 shows the values of the thermodynamic variables obtained from Equations (1) and (2). It is evident from this figure that as the temperature increases, the thermodynamic equilibrium constant (K_c_) decreases, thus confirming that the adsorption process of tequila vinasses compounds is exothermic [26,27]. This is also confirmed by the negative sign of $∆H^{o}$, which indicates an exothermic process. The magnitude of −89.18 kJ mol^−1^ suggests that a chemical adsorption process occurs between the tequila vinasses compounds and the surface of the MaMPT002 adsorbent. The negative value of $∆G^{o}$ is characteristic of a spontaneous and favorable adsorption process. A decrease in randomness at the liquid–solid interface takes place since $∆S^{o}$ shows a negative value. This reveals that the adsorption of tequila vinasses compounds occurs from a disordered phase to an ordered phase on the adsorbent surface. Consequently, the adsorption process of tequila vinasses compounds generates a more orderly rearrangement on MaMPT002 and the loss of the degree of freedom for tequila vinasses compound molecules. The slight entropy loss depicted in Table 5 suggests that the molecules of tequila vinasses compounds were not entirely adsorbed, but they were confined in the liquid contained in the pore spaces in an analogous manner when gas molecules, such as CO_2_, are adsorbed on coals [28].

### 2.4. Approximate Selectivity of the MaMPT002 Adsorbent for Different Tequila Vinasse Compounds

**Figure 4:**
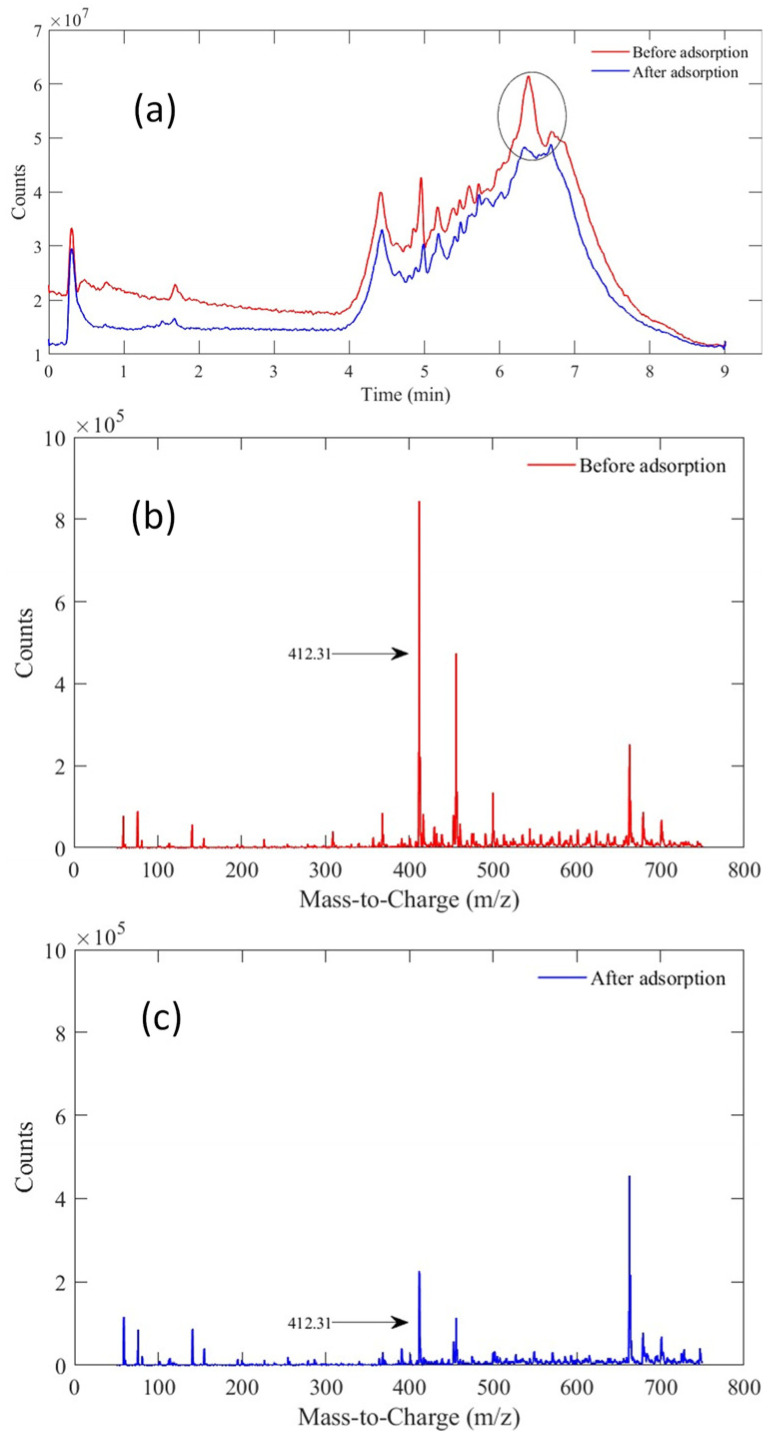
Selectivity results of tequila vinasses adsorption on the MaMPTS002 adsorbent. (**a**) HPLC chromatograms before and after adsorption; (**b**) mass spectrum before adsorption; (**c**) mass spectrum after adsorption.

**Figure 5:**
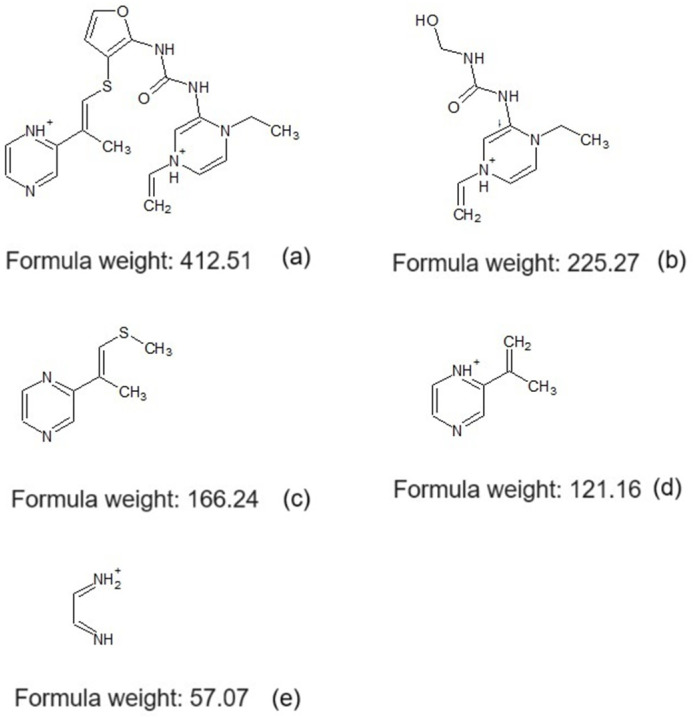
Fragmentation proposal of product ion with 412.31 *m*/*z* (based on the closest structure reported of 412.51 *m*/*z*) obtained from the Optimizer software of the adsorption of tequila vinasses on the MaMPT002 adsorbent.

Chromatograms in Figure 4 indicate a significant intensity decrease in a peak at a time of ~6.4 min after adsorption (indicated by the circle in this figure). This is indicative of the adsorption of compounds present in the tequila vinasses. The mass spectra of Figure 4 indicate a significant decrease in the intensity of most of the signals after adsorption (Figure 4c), except for the signal at ~680 *m*/*z*. This is because the mass spectrometer does not use a standard, but instead it uses the most abundant molecule or fragment as a reference, and the intensity scale is organized around the most abundant fragment with respect to the rest of the fragments. This suggests that the adsorbent shows little selectivity for these molecules. It is worth noting that the complexity of the sample and the large variety of possible molecules corresponding to these masses limit the presentation of one proposal only based on their fragmentations and reported structures. The mass spectrum of Figure 4b shows that the peak of the chromatogram at ~6.4 min corresponds to molecules of 412.31 *m*/*z*. The removal of these molecules is further confirmed by a signal decrease in the mass spectrum after adsorption (Figure 4c). On the other hand, to gain insights about the identity of the molecules being adsorbed, the fragmentation pattern corresponding to the molecules with 412.31 *m*/*z* was used (Table 6). The fragments with a greater probability of coinciding with this mass are shown in Figure 5. According to the results of the software Optimizer, the mass of 412.31 coincides with a type of molecule called melanoidin. This proposal is based on reports by Hofman and Schieberle [15], which included melanoidin structures capable of interacting with thiol groups. Melanoidins are compounds generated by the Maillard reaction which grants a dark brown color to tequila vinasses. Melanoidins show high chelation capacities toward different metal ions and organic compounds. This poses an environmental problem since these properties make them hazardous when discharged to water bodies and fertile lands because they can chelate metallic ions needed for the flora and fauna of the ecosystem.

### 2.5. Physicochemical and Spectroscopic Characterization Results

#### 2.5.1. Textural Properties

**Figure 6:**
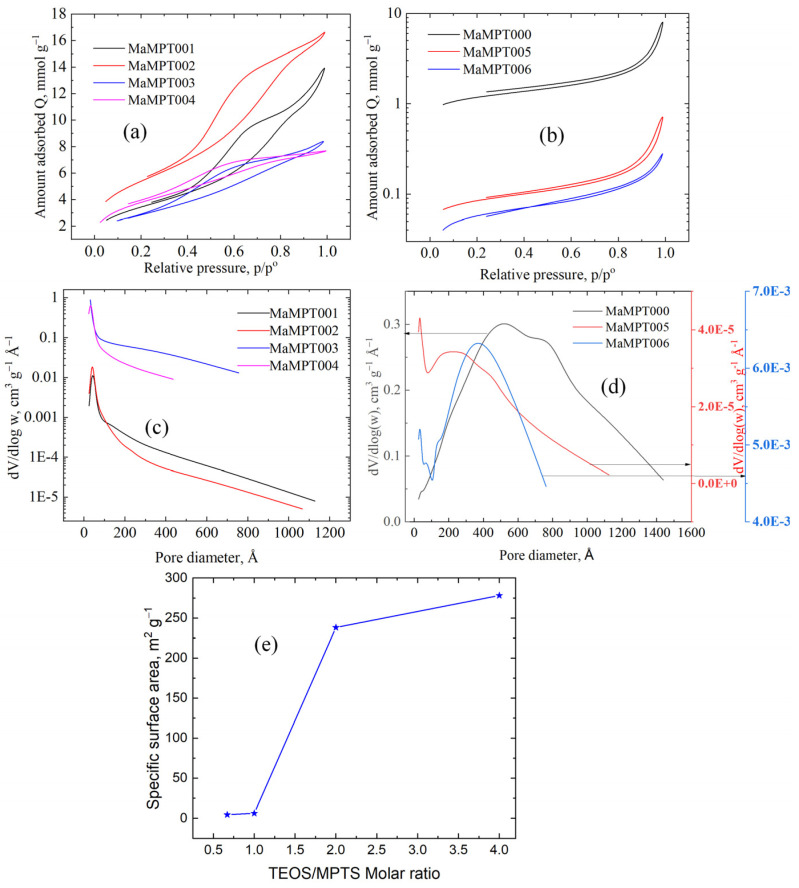
Nitrogen adsorption isotherms and pore size distributions of synthesized adsorbents. (**a**) Isotherms of samples MaMPT001 through MaMPT004; (**b**) isotherms of samples MaMPT000, MaMPT005, and MaMPT006; (**c**) pore size distributions of samples MaMPT001 through MaMPT004; (**d**) pore size distributions of samples MaMPT000, MaMPT005, and MaMPT00, (**e**) TEOS:MPTS molar ratio effect on specific surface area.

Figure 6 shows the N_2_ adsorption isotherms of the synthesized adsorbents. The isotherms of samples MaMPT001-MaMPT004 (Figure 6a) are of the type IVa, conforming to the IUPAC classification [29], and typical of mesoporous materials. In this case, capillary condensation is accompanied by a hysteresis loop that resembles the H2b type more according to this classification, which occurs when the pore width exceeds a given critical size. This type of hysteresis is characteristic of pores with ink-bottle geometries and is often presented by mesocellular silica foams and a few mesoporous ordered silicas obtained after thermal treatment. Some authors have reported that the hysteresis loops start appearing at pore widths of 4 nm [29,30]. This is confirmed in Figure 6b, where the average pore sizes are ~4.2 nm. Samples MaMPT000, MaMPT005, and MaMPT006 exhibit type II isotherms (Figure 6c) and are typical of the physisorption processes on nonporous or macroporous adsorbents. As can be seen from Table 1, these materials presented the lowest specific surface areas. Regarding the pore size distributions, in the case of samples MaMPT001-MaMPT004 (Figure 6b), they depict narrow distributions with a monomodal shape in the mesopore zone of ~2.0–5.0 nm due to the presence of the hysteresis loop observed in the isotherm; a broad part of the distribution is also observed in the region of macropores for these samples. These results imply that adsorbents possessing structures with uniform pores were formed. The sharp slope of the desorption branch of the isotherm proves the uniformity of these pore structures. The opposite situation is obtained in the samples MaMPT000, MaMPT005, and MaMPT006 (Figure 6d), where broad distributions are depicted in the range of pore sizes of ~5.0–145 nm, thus covering a significant amount of the macropores region. A small amount of mesopores is observed in the case of samples MaMPT005 and MaMPT006 represented by a narrow part of the distribution in the zone of ~2.6–4.0 nm.

Different parameters were investigated during the synthesis of adsorbents that can significantly impact the generation of specific surface area, and in the tequila vinasses compounds uptake capacity. These included the following: (1) TEOS:MPTS molar ratios, (2) amount of NaCl, and (3) presence/absence of functionalizing compound (see Table 1). One sample was prepared as described in Section 2.2 but without MPTS. The purpose of synthesizing this sample was to take it as a reference point to compare the effect of the presence of the MPTS molecule on the specific surface area and the tequila vinasse compounds uptake capacities of the adsorbent. A positive effect of obtaining an increased specific surface area can be observed in this table. Also, it is observed that the TEOS:MPTS molar ratio plays an important role in forming specific surface area. In this sense, high TEOS:MPTS molar ratios produce high specific areas, and low TEOS:MPTS molar ratios produce small specific areas. This can be due to the existence of more and greater functional group oligomers, which generate larger cavities around ligand functional groups, which, in turn, contribute to a higher specific surface area (Figure 6e). Another parameter studied was the presence of the stabilizing agent NaCl. The adsorbents MaMPT001 (278.27 m^2^ g^−1^) and MaMPT002 (432.68 m^2^ g^−1^) differ by the presence of NaCl and present a difference in specific surface areas of 154.56 m^2^ g^−1^. Murakata et al. [31] report that the presence of salts significantly affects the formation of the surface area. Some authors attribute this phenomenon to the fact that when a solid surface becomes in contact with an aqueous phase, the surface becomes electrically charged due to the ionization or dissociation of functional groups present on the surface, by the adsorption of ionic groups on the uncharged surface, or the electrochemical polarization. In the case of silanol groups under the presence of water, these groups can be dissociated to form $Si-O^{-}$ in alkaline media or $Si-{OH}_{2}^{-}$ in acidic media. The thickness of the electrical double layer is affected by the electrolytes present and can alter the pore size distribution.

#### 2.5.2. TGA Results

**Figure 7:**
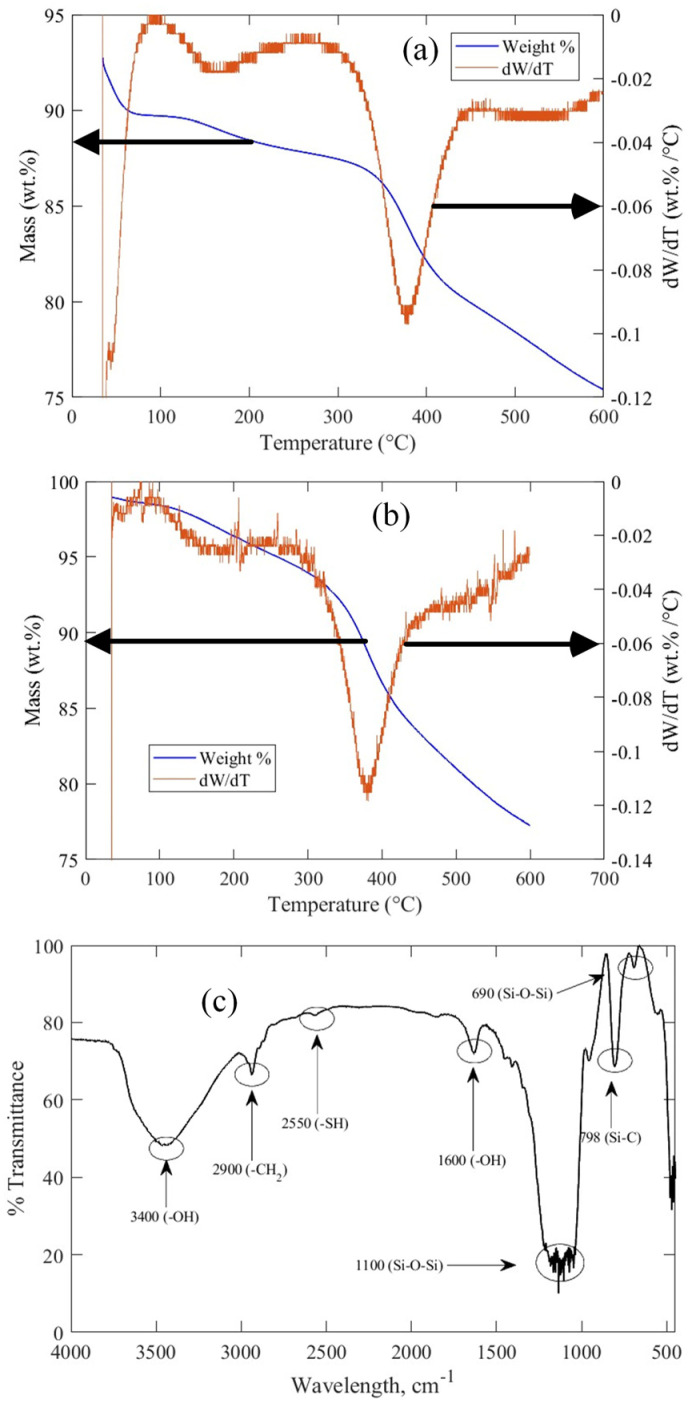
(**a**) TGA results on sample MaMPT002 before tequila vinasses adsorption. (**b**) TGA results on sample MaMPT002 after tequila vinasses adsorption. (**c**) FTIR spectrum of the MaMPT002 adsorbent.

Both thermal stability and thermal changes taking place inside of the MaMPT002 adsorbent were monitored by TGA before and after tequila vinasses removal. According to Almaghrabi et al. [32], the silica matrices present a thermal stability of up to 600 °C. Below 200 °C, a decrease in weight % is observed and associated with the loss of physically adsorbed water (Figure 7a). Above 250 °C, the observed weight % loss is associated with the decomposition of the aliphatic chains of MPTS [33]. A steep slope begins at ~316 °C attributed to the depletion of thiol components [34], constituting about 9.5% of the total mass (corresponding to 3.23% of total sulfur). This result indicates that MaMPT002 adsorbent is thermally stable up to 316 °C. On the other hand, after the tequila vinasses compounds uptake, Figure 7b depicts almost identical weight % loss patterns as before the adsorption of tequila vinasses. In this case, only minor changes are observed in the amount of weight lost and are associated with the adsorbed tequila vinasses. By comparing both graphs, it can be concluded that the thermal stability of the MaMPT002 adsorbent is not affected by the presence of tequila vinasses.

#### 2.5.3. FTIR Study Results

Figure 7c depicts the results of the FTIR analysis of the MaMPT002 adsorbent. Two bands appear at 3400 cm^−1^ and 1600 cm^−1^ and correspond to the stretching and bending of the -OH groups of silanol groups and adsorbed H_2_O, respectively. The signal at 1100 cm^−1^ is due to the stretching vibration of siloxane groups Si-O-Si present in the silica matrix. The bands at 2900, 1400, and 690 cm^−1^ correspond to asymmetric stretching, scissoring movement, and bending of the methylene group CH_2_, respectively, of the MPTS molecule. A signal appears at 798 cm^−1^ corresponding to the Si-C bonds. The functional thiol group (S-H) presents a weak stretching vibration signal at 2550 cm^−1^, and finally, the signal observed at 798 cm^−1^ corresponds to the Si-C bonds.

#### 2.5.4. Scanning Electron Microscopy (SEM)

**Figure 8:**
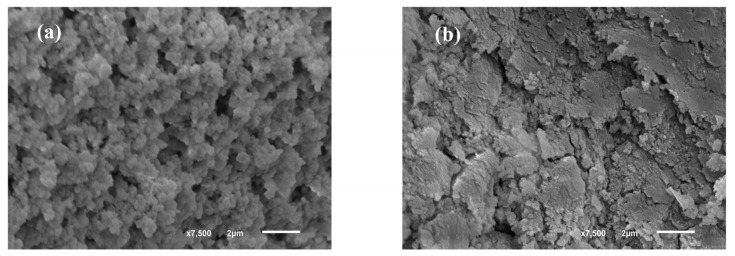
SEM results of (**a**) tequila vinasses-free MaMPT002 adsorbent, and (**b**) MaMPT002 adsorbent after tequila vinasses adsorption.

The structural and morphological changes arising from contacting a tequila vinasses solution with the MaMPT002 adsorbent were investigated by the SEM technique. The results indicate that the surface of the adsorbent comprises a clean and even collection of particles (Figure 8a) before the contact with tequila vinasses. A significant change in the surface is observed after tequila vinasses adsorption, where the adsorbent presents a wrinkled and rugged clustered surface with some flat areas (Figure 8b).

#### 2.5.5. NMR Results

**Figure 9:**
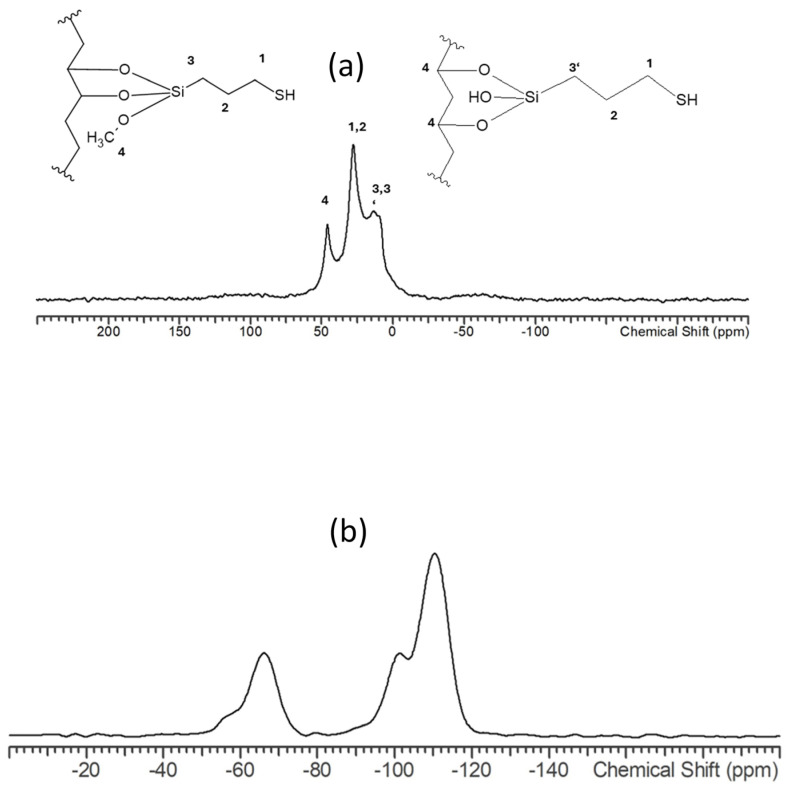
(**a**) ^13^C CP-MAS NMR spectrum of MaMPT002. (**b**) ^29^Si MAS NMR spectrum of MaMPT002.

The propyl thiol-grafted moieties, and the silicon species attached and composing the silica matrix of the MaMPT002 adsorbent, were measured by ^13^C and ^29^Si solid–state NMR spectroscopy, respectively, as shown in Figure 9. In Figure 9a the zone around 12 ppm depicts a broadened signal of two overlapping peaks associated with the carbon C-3 which presents different chemical environments to those of the ligand. Carbons C-1 and C-2 of the MPTS chain are identified by a signal at 28 ppm, and the one corresponding to carbon C-4 of the unreacted and non-hydrolyzed methoxyl groups and present on the matrix silica surface, appears at 45 ppm [18]. On the other hand, in the case of the ^29^Si spectrum (Figure 9b), two main regions of the spectrum are clearly identified, one at −111 ppm and the other one at −63 ppm which correspond, in general, to the Q^m^ and T^m^ species. Particularly, the signals shown in the zone of −111 ppm correspond to the Q^4^ species, and the one at −101 ppm is attributed to a Q^3^ species [18]. This last signal confirms the presence of free silanol groups, SiOH. In addition, the absence of signals at −92 ppm demonstrates the absence of geminal silanols (Si (OH)_2_) in the adsorbent. Additionally, the signals located at −65 ppm correspond to the T^3^ species, whereas the signal at −57 ppm is assigned to the T^2^ species. These results confirm the presence of the ligand groups MPTS to the silica matrix either by two or three covalent bonds, as shown in the schemes of Figure 8a. Furthermore, these results demonstrate that the surface density of the thiol functional groups is higher than that of the residual Q^3^ species, Figure 8b.

## 3. Materials and Methods

### 3.1. Materials

(3-Mercaptopropyl) trimethoxysilane (MPTS) ≥ 99% was used as a functional precursor (FP), and tetraethyl orthosilicate (TEOS) ≥ 99% as a cross-linking agent (CLA); these reagents, sodium chloride, and triethylamine (TEA) ≥99.5%, were all purchased from Sigma Aldrich (Toluca, Mexico). Hydrochloric acid ≥ 70% was purchased from Fermont (Monterrey, Mexico); tequila vinasses were collected from a cooling reservoir in a tequila facility at Amatitan, Jalisco, Mexico.

### 3.2. Synthesis of the Optimum Adsorbent

A set of adsorbents was prepared according to a method described by Lee et al. [35], using the sol–gel modified process. In a typical synthesis, the FP and CLA were hydrolyzed and partially homocondensed by mixing each separately with deionized H_2_O, NaCl, and EtOH. All reactions were performed at a pH of 3 adjusted with 3.0 M of HCl dropwise (used as a catalyst). The FP and CLA solutions were stirred for 30 min at room temperature. Afterward, the FP and CLA solutions were mixed and stirred for another 30 min to allow the co-condensation of the silanes. TEA was added to the mixture to induce gelation of the system. The obtained gel was aged for 24 h at room temperature and dried in an oven at 60 °C for 24 h. After that, the obtained material was subjected to acetone/water washings at 60 °C. A drying process was repeated at 60 °C for 24 h. Finally, the adsorbents were ground and sieved to a particle size of 125–180 µm to minimize the external mass transfer resistances, as reported elsewhere [36,37]. Different molar ratios of FP:CLA were studied during the synthesis to select the optimum hybrid material from the set of adsorbents, which had the largest specific surface area and the highest tequila vinasses removal capacity. The optimum adsorbent was used for full characterization. The adsorbents were identified as MaMPTXX where XX corresponds to the test number conducted at the different synthesis conditions. In a preliminary experiment, the seven adsorbents synthesized were assessed for the maximum tequila vinasses adsorption from a 0.033 mg mL^−1^ tequila vinasses stock solution. The results of this experiment indicated that the highest uptake of 0.80 g g^−1^ was obtained from the MaMPPT002 adsorbent, which can be attributed to its high specific surface area (Table 1). This adsorbent was used for the subsequent characterization.

### 3.3. Analysis Procedure of Tequila Vinasses Color Solution

The colored solutions have been studied using several analyses based on the Lambert–Beer law. It is well known that because tequila vinasses are a complex mixture of chemical compounds, they present complicated UV-vis absorption profiles due to the set of different molar extinction coefficients, thus displaying several peaks within a wide range of wavelengths. This presents a problem in assessing the accurate concentration of tequila vinasses for a given component. To overcome this problem, in this work, a procedure is proposed whereby the integration of the areas under the curve of UV-vis signals (using a Thermo scientific Genesys 150 UV–visible spectrophotometer, Waltham, Massachusetts, USA) of a full scan spectrum of wavelengths between 350 nm and 900 nm, were related to the drained mass of the solution using different vinasse concentrations. A calibration curve of the weight of the solids/mL vs. total absorbance was constructed by first placing an accurately measured fixed volume of tequila vinasses (2 mL ± 0.01 mL) in two sets of 4 ceramic crucibles, each at different vinasse:water volume ratios (2:0; 1.5:0.5; 1:1; 0.5:1.5; 0:2 mL of tequila vinasses mL^−1^ of water). The first set of crucibles was dried until constant weight in a vacuum oven at 50 °C. After that, the total dry weight was registered. This weight was then divided by the original volume of tequila vinasses used so that the amount of the solids of vinasses per mL was obtained and expressed in g of solids mL^−1^ of vinasses. For each crucible of the second set, a full UV-vis scan was performed in the range of 350–900 nm. The whole spectrum was numerically integrated using Simpson’s 1/3 rule (see Table S1 of the Supplementary Materials) to obtain the corresponding absorbance of all compounds present in the tequila vinasses for each point of the calibration curve. In the case of all adsorption experiments (kinetics, and isotherms fixed bed), the previously diluted samples were analyzed by performing the full scan in the UV-vis to find the corresponding total absorbance and referred to the calibration curve to determine the concentration.

### 3.4. Kinetics of Vinasses Compounds Adsorption Experiments

Prior to the experiments, the solution of tequila vinasses was filtered and centrifuged to remove the suspended solids and other components. Three different tequila vinasses concentrations (0.0052, 0.0137, and 0.0275 g mL^−1^) were used to study the effect of the initial solution concentration on the adsorption rate. In total, 20 mg of the MaMPT002 adsorbent and 2 mL of the vinasses solution at a pH of 3.55 were contacted in Eppendorf tubes at varying times (5–200 min) and stirred at 25 °C in a temperature-controlled bath. After adsorption, the final suspensions were filtered, and the collected solutions (final solutions) were measured for tequila vinasses concentrations. Three replicate experiments were conducted for each curve. The amount of tequila vinasses compounds adsorbed was calculated by Equation (4):

(4) $$q=\;\frac{V\;\left(C_{i}-C_{f}\right)}{m}$$

where *q* (g g^−1^) is the amount of tequila vinasses compounds adsorbed per mass unit of adsorbent at a given time *t*, *C_i_* and *C_f_* (g L^−1^) are the initial and final solution of tequila vinasses concentrations, respectively; V (L) is the solution volume, and *m* (g) is the mass of adsorbent.

### 3.5. Vinasses Compounds Adsorption Isotherm Experiments

The adsorption equilibrium of tequila vinasses on the MaMPT002 adsorbent was investigated using the batch mode to evaluate the maximum uptake capacity of adsorbent at three different pH values (3.0, 3.55, and 4.0) common of the vinasse discharges from tequila industries [17]. Tequila vinasses solutions at varying initial concentrations (0.031–0.097 g mL^−1^) and fixed initial pH were placed in contact in Eppendorf tubes with 20 mg of adsorbent and stirred for 90 min in the temperature-controlled bath at 25 °C. The suspensions were filtered, and the initial and final tequila vinasses solution concentrations were measured. The amount of vinasses compounds adsorbed were calculated by Equation (1). The values reported were the averages of three replicate experiments.

### 3.6. Selectivity of MaMPT002 for Tequila Vinasses Compounds

Tequila vinasses are a complex mix of more than two hundred diverse types of compounds (e.g., monosaccharides, VFA, alcohols, minerals, melanoidins, among other compounds) [38]. Therefore, due to the difficulty of identifying and quantifying each compound that is adsorbed on the surface of the adsorbent, an approximate qualitative measurement was implemented in this work. To this aim, a tequila vinasses solution (10 mL, initial solution) was contacted with the MaMPT002 adsorbent (5 g) for 24 h at 25 °C to assess the selectivity of the adsorbent for the different tequila vinasses compounds. The resulting suspension was filtered, and the collected solution was called the final solution. Then, the initial and final solutions were diluted 1/100 and analyzed by HPLC-MS/MS. By observing the differences in the presence/absence of the characteristic signals of the spectra and chromatograms before and after tequila vinasses adsorption, one can gain preliminary insights about the type of compound(s) being removed and qualitatively assess the affinity/selectivity of the adsorbent for a group of compounds interacting with the solid. An Agilent Technologies 1200^®^ instrument coupled to a 6430B Mass Spectrometer was used. A C18 Zorbax Eclipse XDB column (50 mm × 2.1 mm × 3.5 μm) was used. The mobile phase was 0.1% *v*/*v* of formic acid in water and acetonitrile gradient, starting with a ratio of 90% water and 10% acetonitrile, which after 5 min ended up in a ratio of 10% water and 90% acetonitrile, at a rate flow of 0.5 mL min^−1^. The fragmentation of the molecule of interest was identified with the aid of software Optimizer V9.09 from Agilent Technologies Company (Santa Clara, CA, USA).

### 3.7. Physicochemical and Spectroscopic Characterization of the Adsorbent

#### 3.7.1. Textural Properties of Adsorbent

Textural properties of the MaMPTXX adsorbents were measured by N_2_ adsorption–desorption experiments at 77 K using a $N_{2}$ adsorption equipment ASAP 2020 KMP Micromeritics (Norcross, GA, USA). Preceding the measurements, about 0.2 g of the samples were conditioned at 423 K and a vacuum of 10 µmHg for 12 h to detach all possible species adsorbed on the surface of the adsorbent, such as water and CO_2_ and all that could interfere with the measurement. The specific surface area was determined by the Brunauer–Emmett–Teller (BET) method using adsorption data in the relative pressure range of 0 < P/$P_{o}$ < 0.3, the total pore volume was determined by adsorption at P/$P_{o}$ = 0.995, and the pore size distribution was obtained by the Barrett–Joyner–Halenda (BJH) method.

#### 3.7.2. Thermogravimetric Analysis (TGA)

The thermal stability of the MaMPT002 adsorbent was studied by TGA. Dried samples were analyzed using a Discovery model analyzer from TA Instruments (Amarillo, TX, USA) using dried samples. About 10–15 mg of the sample was placed in a platinum pan; a temperature scanning range of 25–600 °C was used; the heating rate was 10 °C min^−1^ under a nitrogen atmosphere at 60 mL min^−1^.

#### 3.7.3. Fourier Transform Infrared Measurements (FTIR)

The thiol functional groups on the surface of the adsorbent were investigated by FTIR using the KBr method. A KBr salt and an amount of adsorbent were dried at 333 °K, mixed, and compressed to obtain a KBr pressed disc. FTIR spectra were obtained with a FTIR Spectrum One Perkin Elmer instrument equipped with an IR source and a KBr splitter. The spectra of the samples were obtained using 64 scans from 4000 to 500 cm^−1^ and a resolution of 4 cm^−1^.

#### 3.7.4. Scanning Electron Microscopy (SEM) Study

Changes in the textural properties and morphology of the MaMPT002 adsorbent before and after tequila vinasses adsorption were studied using the SEM technique. A JEOL JSM6610LV microscope (Akishima, Tokyo, Japan) was employed and operated at 15 kV at 14.92–15.02 mm using a gold coating for 20 s.

#### 3.7.5. Solid–State NMR Spectroscopy

^13^C CP-MAS NMR was used to observe the carbon presence of the MPTS ligand groups on the adsorbent; ^29^Si MAS NMR was conducted to obtain information about the structure of the silicon matrix, and the grafting degree of the adsorbent through the Q and T species. Preceding all experimental measurements, samples were desiccated during 48 h at 333 K. Solid–state NMR assessments were conducted at ambient temperature using an Infinityplus spectrometer apparatus (Agilent) maintained under a magnetic field of 7 T and equipped with a changing temperature Chemagnetics–Varian 6 mm pencil cross-polarization (CP) magic angle spinning (MAS) probe. ^13^C {^1^H} CP MAS NMR spectra were acquired using a cross-polarization contact time of 3 ms, a 90° pulse with a length of 5.0 μs, a relaxation delay of 5 s, with MAS at 7 kHz. ^29^Si{^1^H} CP MAS NMR spectra were acquired using a cross-polarization contact time of 5 ms, a 90° pulse with a length of 5.0 μs, a relaxation delay of 1 s, under MAS at 7 kHz. In general, the advantage of the cross-polarization method in significantly increasing the signal-to-noise ratio is partially offset by the fact that the signal intensity is no longer proportional to the concentration of the corresponding structural units in the sample. This means that the ratio between the Q^3^ and Q^4^ units cannot be measured using ^29^Si{^1^H} CP NMR. Such a measurement would require a very long measurement time [39,40]. However, if the cross-polarization contact time is long enough, the ratio between Q^2^, Q^3^, T1, and T^2^ units can be measured quite accurately [41,42].

## 4. Conclusions

A propyl thiol-functionalized silica gel adsorbent was synthesized and applied in the removal of tequila vinasses color compounds from tequila industries. Due to the difficulty of unambiguously evaluating the amounts and types of tequila vinasses components adsorbed, a simple analysis procedure was proposed and applied to analyze tequila vinasses concentrations. The removal of tequila vinasses achieved a maximum uptake of 0.801 g g^−1^ at a pH of 3.55. The adsorption of tequila vinasses demonstrated rapid kinetics (~90 min). The kinetic data matched the PFO model. The adsorption equilibrium isotherm data were explained by the Temkin model. The MaMPT002 adsorbent revealed that vinasses uptake capacities were enhanced/comparable to adsorbents possessing diverse complexing functional groups found in other published works. The MaMPT002 adsorbent showed preferential reactivity toward melanoidin-type compounds, as observed in the HPLC-MS/MS study. A plausible approach to the identity of the removed melanoidin-type compound with 412.31 *m*/*z* was proposed. The textural properties of the MaMPT002 adsorbent include enhanced specific surface areas and pore sizes suitable for the easy diffusion of tequila vinasses molecules to the adsorption sites. TGA results indicated that this adsorbent is thermally stable up to 316 °C and the presence of tequila vinasses did not affect its thermal stability. FTIR and solid–state NMR results confirmed the chemical functionalization and the matrix solid structure of the MaMPT002 adsorbent. The results of this work suggest that such adsorbents can be effective in treating tequila vinasses compounds from industrial wastewater. The present work represents the first integral approximation to the development of selective functionalized material to adsorb melanoidin-type compounds from the vinasses of the tequila industry as well as the evaluation of its efficiency as adsorbent in packed-bed systems.

## Figures and Tables

**Table 1:** Molar ratios used in the synthesis of thiol-functionalized adsorbents for the treatment of tequila vinasses.

Adsorbent	Molar Ratios									TEOS Hydrolysis Time, Min	TEOS/MPTS Co-Condensation Time, Min	Mixture pH	S_BET_, m^2^ g^−1^	Avg. Pore Diameter D_p_, Å	q_max_ *^a^*, mmol g^−1^
	TEOS Mixture				MPTS Mixture				TEA						
	TEOS	NaCl	EtOH	H_2_O	MPTS	NaCl	EtOH	H_2_O							
Ma000	1	0.009	4	4	0	0	0	0	0.09	30	30	3.0	88.0	203.07	−
MaMPT001	1	0.009	4	4	0.25	0	3	1	0.12	30	30	3.0	278.27	56.69	−
MaMPT002	1	0.009	4	4	0.25	0.009	3	1	0.12	30	30	3.0	432.68	47.19	1.01
MaMPT003	1	0.009	4	4	0.5	0	3	1	0.12	30	30	3.0	238.12	39.29	−
MaMPT004	1	0.009	4	4	0.5	0.009	3	1	0.12	30	30	3.0	311.18	34.10	−
MaMPT005	1	0.009	4	4	1	0	3	1	0.12	30	30	3.0	6.44	211.30	−
MaMPT006	1	0.009	4	4	1.5	0	3	1	0.12	30	30	3.0	4.58	104.10	−

*^a^* Maximum uptake capacity of adsorbent measured by elemental analysis as total sulfur.

**Table 2:** Results of regressed parameters of the adsorption kinetic models of tequila vinasses compounds on the MaMPT002 adsorbent.

**Parameters/Model**	**Initial Concentration of Tequila Vinasses Compounds,** **g mL^−1^**		
	0.0275	0.0137	0.0052
q_max exp_ (g g^−1^)	0.79	0.47	0.17
	Pseudo-first order		
q_max calc_ (g g^−1^)	0.61 ± 0.04	0.51 ± 0.02	0.19 ± 0.02
*k*_1_ (min^−1^)	0.2 ± 0.1	0.14 ± 0.03	0.016 ± 0.003
R^2^	0.6474	0.9004	0.9527
	Pseudo-second order		
q_e calc_ (g g^−1^)	0.68 ± 0.06	0.53 ± 0.03	0.27 ± 0.04
*k*_2_ (g g^−1^ min^−1^)	0.3 ± 0.2	0.5 ± 0.3	0.05 ± 0.02
R^2^	0.7447	0.8587	0.9429
	Intraparticle diffusion		
*K*_IPD_ (g g^−1^) × min^−1/2^)	0.18 ± 0.03	0.14 ± 0.03	0.01 ± 0.01
C (g g^−1^)	0.08 ± 0.07	0.10 ± 0.08	−0.06 ± 0.03
R^2^	0.8401	0.6476	0.7981

**Table 3:** Fitting results of the adsorption isotherm models to experimental data of tequila vinasses adsorption on the MaMPT002 adsorbent.

**Parameters/Model**	**Initial pH of Tequila Vinasses Solution**		
	3.0	3.55	4.0
q_max exp_ (g g^−1^)	0.78	0.80	0.77
	Langmuir		
q_max calc_ (g g^−1^)	0.99 ± 0.05	1.3 ± 0.2	1.2 ± 0.3
*k*_1_ (min^−1^)	147 ± 20	70 ± 25	95± 46.5
R^2^	0.9740	0.9285	0.8430
	Freundlich		
*k_F_* (L^n^ mg^−n^)	3.2 ± 0.7	6.5 ± 2.9	5.5 ± 3.0
*n*	2.7 ± 0.3	1.8 ± 0.3	2.0 ± 0.5
R^2^	0.9306	0.8808	0.7744
	Temkin		
*A_T_*	1302 ± 297	557 ± 110	667 ± 229
*b_t_* (J mol^−1^)	10948 ± 947	7834 ± 840	8307 ± 1534
R^2^	0.9642	0.9461	0.8556

**Table 5:** Thermodynamic parameters obtained by the non-linear regression of Equation (4).

T (K)	K_c_ (mL g^−1^)	K_c_	ΔS^°^ (kJ/mol)	ΔH^°^ (kJ/mol)	ΔG^°^ (kJ/mol)
−	−	−	−0.266	−89.18	−
298	121.64	55.48	−	−	−10.02
303	75.27	34.33	−	−	−8.69
308	51.94	23.69	−	−	−7.36
313	0.35	0.16	−	−	−6.04

**Table 6:** Fragmentation pattern result of the molecular ion with 412.31 *m*/*z* from the Optimizer software of the adsorption process of tequila vinasses on the MaMPT002 adsorbent.

The Final Product Ion, *m*/*z*	Abundance
227.1	92341
165.1	13044
121.1	27677
57.2	18350

## Data Availability

Data are contained within the article and Supplementary Materials.

## Supplementary Materials

The following supporting information can be downloaded at: https://www.mdpi.com/article/10.3390/molecules29245910/s1, Supplementary S1. Calculation example of the area under the curve of the UV-vis scanning spectra of tequila vinasses using Simpson’s 1/3 rule. Supplementary S2. Description of kinetics, isotherms adsorption models. Supplementary S2.1. Adsorption kinetic models [43,44,45]. Supplementary S2.2. Adsorption isotherm models [46].
