# Supplementary material for: Color Compounds Removal from Tequila Vinasses Using Silica Gel Adsorbents Functionalized with Thiol Moieties: Equilibrium and Kinetics Studies
Source: Molecules. 2024 Dec 15;29(24):5910. doi: 10.3390/molecules29245910 (PMC11679727; doi:10.3390/molecules29245910)
Supplement: Supplementary file 1 [file molecules-29-05910-s001.zip › molecules-3276230-supplementary.pdf]

Supplementary Materials

S1. Calculation example of the area under the curve of the UV-vis scanning spectra of tequila vinasses using Simpson´s 1/3 rule.

In this work, the Simpson´s 1/3 rule was used to numerically integrate the measured % absorbances data of the UV-vis spectra and further employed to calculate the tequila vinasses concentrations of all adsorption experiments. Table S1 shows an example with some measured % absorbances at different wavelengths for the calculation of the tequila vinasses concentrations of experiment XX.

The Simpson´s rule 1/3 equation is given by the following:

$$\int_a^b f(x)\,dx \approx S_n = \frac{\Delta x}{3}\left[f(x_0) + 4f(x_1) + 2f(x_2) + 4f(x_3) + \ldots + 2f(x_{n-2}) + 4f(x_{n-1}) + f(x_n)\right] \quad (1)$$

where *f(x)* represents the experimentally measured % absorbances through the whole range of UV-vis scanning, x is a wavelength single point (nm), $\Delta x = \frac{(b-a)}{n}$, where *b* is the upper limit of integration (*b* = 900 nm), a is the lower limit of integration (*a* =325 nm), and *n* is the total number of points in which the interval was divided (*n* = 900–325= 575 points).

Table S1. Simpson's 1/3 rule for the calculation of the area under the curve of the UV-vis scanning spectra of tequila vinasses.

Wavelength λ, nm	$f(x_n)$ = % absorbance
900	0.00676
899	0.00834
898	0.00863
897	0.0093
327	0.67458
326	0.68489
325	0.69581

S2. Description of Kinetic and Isotherm Models

The parameters of all models were obtained by nonlinear regression of the data using the Levenberg–Marquardt algorithm. All modeling calculations were carried out using MATLAB ® software.

S2.1 Adsorption kinetic models

S2.1.1 Pseudo-First Order (PFO) model

The PFO model has been used to describe several kinetic processes. The model assumes that when two phases (solid and liquid) get in touch with each other, a reversible reaction occurs and a dynamic equilibrium linking the two phases is set in the adsorption operation; the model also assumes that the adsorbate accumulates on the adsorbent surface as time proceeds (Chen, 2009). The PFO model is represented by Eq (2):

$$q_t = q_e(1 - e^{-k_1 t}) \tag{2}$$

where q_t $(mg\ g^{-1})$ is the amount of tequila vinasses adsorbed at a given time t (min), q_e $(mg\ g^{-1})$ is the maximum adsorption capacity of tequila vinasses at equilibrium, and k_1 (min^{-1}) is the rate constant of the PFO.

S2.1.2 Pseudo-Second Order (PSO) model

The PSO model was derived under the assumption that proportionality exists between the reaction rate and the adsorption capacity at equilibrium. It establishes that the adsorption rate is proportional to the active sites and the total amount of adsorbed difference (Ho and McKay, 1998). The PSO model is represented by Eq (3):

$$q_t = \frac{q_e^2\, k_2\, t}{1 + q_e\, k_2\, t} \tag{3}$$

where $k_2 (mg\ g^{-1}\ min^{-1})$ is the rate constant of the PSO.

S2.1.3 Intraparticle diffusion (IPD) model

The IPD model was presented by Weber and Morris (Weber and Morris, 1963). In this model, attainment of the dynamic equilibrium can be restricted by intraparticle diffusion of tequila vinasses over and/or within the particle of MaMPT002. It is often used to describe adsorption kinetics of large particle size and uniform pores. If the line goes by the origin point (0, 0), the mechanism is controlled by the intraparticle diffusion; contrarily, it is a multiple adsorption process (intraparticle diffusion and external film). The IPD model is represented by Eq (4):

$$q_t = K_{IPD}\, t^{1/2} + C \tag{4}$$

where $K_{IPD} (mg\ g^{-1}\ min^{-1/2})$ is the rate constant of the IPD model, and C (g g^{-1}) is a constant.

S2.2. Adsorption Isotherm Models

Langmuir. This model was derived assuming that the adsorption occurs on a finite number of energetically homogeneous binding sites and one adsorbate molecule binds to one binding site (Foo and Hameed, 2010). *The Freundlich model* describes a non-ideal reversible adsorption and is not restricted to monolayer

formation. This empirical model assumes a multilayer for heterogeneous adsorption sites (Foo and Hameed, 2010). *The Temkin model* was derived assuming that the heat of adsorption (ΔH_{ads}) of all molecules in the layer decreases linearly because of surface coverage. Also, the model considers the effects of indirect adsorbent–adsorbate interactions on the adsorption process. The equations describing these models are shown below:

Langmuir:

$$q_e = \frac{q_{max}\, K_L\, C_e}{1 + K_L\, C_e} \tag{5}$$

Freundlich:

$$q_e = K_f C_e^{1/n} \tag{6}$$

Temkin:

$$q_e = \frac{RT}{b_T} Ln\, A_T C_e \tag{7}$$

where q_e ($g\ \ g^{-1}$), represents the amount of tequila vinasses compounds adsorbed at equilibrium; q_{max} ($g\ g^{-1}$) is the maximum amount of adsorbate; C_e ($g\ mL^{-1}$) is the concentration at equilibrium of the tequila vinasses solution; K_L ($mL\ g^{-1}$) is the Langmuir model constant which is related to adsorption affinity; K_f ($L^n\ mg^{-n}$) and n are Freundlich model constants; b_T ($J\ mol^{-1}$) is the Temkin constant which is related to the heat of adsorption; A_T ($L\ g^{-1}$) is the Temkin isotherm constant; T(K) is the temperature of the process; and R ($J\ K^{-1} mol^{-1}$) is the gas constant.

References

1. Chen, H.; Wang, A. Adsorption characteristics of Cu(II) from aqueous solution onto poly(acrylamide)/attapulgite composite. *J. Hazard. Mater.* **2009**, *165*, 223–231. https://doi.org/10.1016/j.jhazmat.2008.09.097.
2. Ho, Y.S.; McKay, G. A Comparison of Chemisorption Kinetic Models Applied to Pollutant Removal on Various Sorbents. *Process Saf. Environ. Prot.* **1998**, *76*, 332–340. https://doi.org/10.1205/095758298529696.
3. Weber, W.J.; Morris, J.C. Kinetics of Adsorption on Carbon from Solution. *J. Sanit. Eng. Div.* **1963**, *89*, 31–59. https://doi.org/10.1061/JSEDAI.0000430.
4. Foo, K.Y.; Hameed, B.H. Insights into the modeling of adsorption isotherm systems. *Chem. Eng. J.* **2010**, *156*, 2–10. https://doi.org/10.1016/j.cej.2009.09.013.
